# Interpretation of HRCT Scans in the Diagnosis of IPF: Improving Communication Between Pulmonologists and Radiologists

**DOI:** 10.1007/s00408-018-0143-5

**Published:** 2018-08-10

**Authors:** Jonathan H. Chung, Jonathan G. Goldin

**Affiliations:** 1 Department of Radiology, The University of Chicago Medical Center, 5841 S. Maryland Avenue, MC 2026, Chicago, IL 60637 USA; 2 Department of Radiology, David Geffen School of Medicine at UCLA, Santa Monica, CA USA

**Keywords:** Interstitial lung disease, Idiopathic pulmonary fibrosis, Diagnostic criteria, Multidisciplinary discussion, High-resolution computed tomography

## Abstract

Idiopathic pulmonary fibrosis (IPF) is a progressive fibrosing interstitial lung disease (ILD). In this review, we describe the central role of high-resolution computed tomography (HRCT) in the diagnosis of IPF and discuss how communication between pulmonologists and radiologists might be improved to make the interpretation of HRCT scans more effective. Clinical information is important in the interpretation of HRCT scans, as the likelihood that specific radiologic features reflect IPF is not absolute, but dependent on the clinical context. In cases where the clinical context or HRCT pattern are inconclusive, multidisciplinary discussion (MDD) between a pulmonologist and radiologist (and, where relevant, a pathologist and rheumatologist) experienced in the differential diagnosis of ILD is necessary to establish a diagnosis. While it can be challenging to convene a face-to-face meeting, MDD can be conducted virtually or by telephone to enable each specialty group to contribute. To make the MDD most effective, it is important that relevant clinical information (for example, on the patient’s clinical history, exposures and the results of serological tests) is shared with all parties in advance. A common lexicon to describe HRCT features observed in ILD can also help improve the effectiveness of MDD. A working diagnosis may be made in patients who do not fulfill all the diagnostic criteria for any specific type of ILD, but this diagnosis should be reviewed at regular intervals, with repeat of clinical, radiological, and laboratory assessments as appropriate, as new information pertinent to the patient’s diagnosis may become available.

## Introduction

Idiopathic pulmonary fibrosis (IPF) is a chronic and ultimately fatal interstitial lung disease (ILD) characterized by progressive fibrosis and loss of lung function [1]. IPF is a rare disease and mainly affects older adults, with data from a US healthcare insurance claims database suggesting an incidence of 19.3 per 100,000 person-years in individuals aged 55–64 years [2]. Historical data suggest a median time from diagnosis of IPF to death of only 2‒3 years [1], but survival time following diagnosis is likely improving as patients are diagnosed earlier and are treated with drugs that slow disease progression [3–6].

Prompt diagnosis of IPF is important to ensure that patients receive appropriate care and support and have the opportunity to receive anti-fibrotic therapy and be evaluated for lung transplantation. Accurate diagnosis of IPF is critical, as other forms of ILD that have similar clinical presentations to IPF require different treatment strategies [7–11]. Imaging plays an essential role in the diagnosis of IPF [1]. Once known causes of ILD have been excluded, a usual interstitial pneumonia (UIP) pattern on high-resolution computed tomography (HRCT) is essentially diagnostic of IPF in the appropriate clinical setting [1, 12]. In addition, some non-UIP HRCT patterns strongly suggest an alternative diagnosis [12]. Multidisciplinary discussion (MDD) between pulmonologists, radiologists and, where appropriate, pathologists and rheumatologists experienced in the diagnosis of ILD is key to establishing the diagnosis of IPF [1, 12, 13]. However, the relay of information between radiologist and pulmonologist may be hampered by busy clinical schedules or misaligned expectations. In this review, we describe the central role of HRCT scans in the diagnosis of IPF and how clinical information provided by pulmonologists and other clinicians can aid radiologists in the interpretation of HRCT scans.

## HRCT Scans in the Diagnosis of IPF

Central to the diagnosis of IPF is the performance and interpretation of an HRCT scan [1]. A surgical lung biopsy may be warranted if the HRCT pattern is inconclusive and the benefit of obtaining a more confident diagnosis outweighs the risks of conducting a biopsy in that patient [12]. Current international guidelines for the diagnosis of IPF, published in 2011, state that the HRCT criteria for a definite UIP pattern characteristic of IPF are predominantly subpleural, basal reticular abnormalities in the presence of honeycombing, with or without traction bronchiectasis, and the absence of any of inconsistent features [1]. Inconsistent features include: upper or mid-lung predominance, peribronchovascular predominance, extent of ground-glass abnormality greater than reticular abnormality, profuse micronodules (bilateral, predominantly upper lobes), discrete cysts (multiple, bilateral, away from areas of honeycombing), diffuse mosaic attenuation/air-trapping (bilateral, in three or more lobes), or consolidation in bronchopulmonary segment(s)/lobe(s). When performed correctly in the relevant patient population, UIP on HRCT has a high specificity for IPF [14–16]. However, it is important to note that our knowledge of radiological features that are diagnostic and characteristic of IPF comes from studies based on high-quality HRCT scans, not from more rudimentary radiographs. The classic studies that informed our knowledge of HRCT features in patients with IPF utilized protocols in which thin sections at 1–3 cm intervals were obtained using collimation sections of 1.0–3.0 mm and were reconstructed using a high-spatial-frequency algorithm [17–22], although current standard of care is to acquire images with volumetric CT and then to reconstruct at this slice reconstruction. In clinical practice, HRCT scans also need to be high-quality to ensure that key radiological features that are characteristic of IPF, or that would argue against a diagnosis of IPF, are apparent.

Current international guidelines for the diagnosis of IPF include recommendations for the optimal HRCT technique for evaluation of ILD [1]. These include that non-contrast axial scans should be obtained in the supine position unless dependent density obscures detail, in which case prone scans can be used (with the option of coronal and sagittal reconstructions if volumetric images are obtained). In many expert centers, both supine and prone images are acquired, and this is becoming (and some argue, has become) standard of care. The field of view should include only the lungs to maximize in-plane spatial resolution. Scans should be obtained during full inspiration without respiratory motion. An expiratory scan is helpful to exclude lobular air-trapping suggestive of hypersensitivity pneumonitis or connective tissue disease and is a mandatory part of the HRCT protocol for evaluation of ILD. Thin sections (≤ 2 cm intervals) can be contiguous or non-contiguous and images should be reconstructed using a high-spatial-frequency reconstruction algorithm. Though not essential, coronal and sagittal reformations can be helpful in determining the distribution of disease as well as identifying subtle honeycombing.

## The Importance of Multidisciplinary Discussion in Differential Diagnosis of ILD

MDD involving a radiologist, a pulmonologist and, when necessary, a pathologist, with experience in the differential diagnosis of ILD is the gold standard in the diagnosis of IPF [1, 12, 13] and has been shown to improve the confidence of the diagnosis [23, 24]. In a classic study in which clinicians, radiologists, and pathologists were provided with clinical, HRCT, and histopathological data from 58 cases of suspected idiopathic interstitial pneumonia in a stepwise fashion, interdisciplinary agreement improved as more data were provided and the case was discussed [23]. In a recent study in which data from 70 patients with diffuse parenchymal lung disease were evaluated by seven multidisciplinary teams, MDD was associated with the assignment of a diagnosis of IPF with high confidence more frequently than when diagnoses were made by clinicians or radiologists alone; further, supporting the validity of the diagnoses made following MDD, there was a greater separation in the mortality rates of patients with an IPF versus non-IPF diagnosis made by the multidisciplinary teams than by clinicians or radiologists alone [24].

A white paper recently published by the Fleischner Society [12] proposed that not all patients with suspected IPF require MDD. Rather, MDD is necessary when the clinical context and/or HRCT pattern are inconclusive (to determine additional clinical evaluations), after biopsy (to integrate clinical, imaging, and histological findings), to consider a working diagnosis in patients who do not fulfill all the diagnostic criteria for any specific type of ILD (i.e., who are not adequately covered by the existing evidence base), and to revisit cases where the disease course is discordant with the established diagnosis. When a working diagnosis of IPF is made, the patient should be closely monitored, with repeat of clinical, radiological, and laboratory assessments as appropriate, so that their diagnosis can be reviewed at regular intervals (ideally based on further rounds of MDD).

While it can be challenging to convene a multidisciplinary team face-to-face, MDD can be conducted virtually or by telephone to enable each specialty group to contribute. To make the MDD most effective, comprehensive clinical and HRCT data should be made available in advance of the MDD so that all parties can consider the case in advance. A common understanding among all parties of the terms used to describe HRCT features observed in ILD [25] can also help improve the effectiveness of MDD. When IPF is considered in the differential diagnosis, the radiologist should advise whether a UIP pattern is present and, if so, their level of confidence, based on evaluation of image quality and the distribution and extent of specific disease features defined based on standard terminology. The identification of a UIP pattern can be more challenging in patients (usually ex-smokers) who have both lung fibrosis and emphysema. In such cases, radiologists should describe the extent and severity of emphysema as well as UIP, as this may influence patient management, evaluation and prognosis. It is important that the outcome of the MDD is adequately documented, including the first choice diagnosis (which may be “unclassifiable disease”), realistic differential diagnoses, likely reversibility of the patient’s disease, and recommendations on additional diagnostic tests [12].

## Clinical Information to Aid the Interpretation of HRCT Scans

Clinical information is key to the interpretation of HRCT scans, as the likelihood that specific radiologic features reflect IPF is not absolute, but dependent on the clinical context. Indeed, making a diagnosis of IPF specifically requires the exclusion of known causes of ILD, including autoimmune diseases, exposure to potential inducers of chronic hypersensitivity pneumonitis, occupational exposures, and the use of certain drugs [26]. To assist the radiologist in contributing to the MDD, it is important that they have access to relevant information on the patient’s clinical history, exposures and the results of other tests that have been performed (Table 1). While the radiologist does not need to know every detail, if the pulmonologist or other clinician involved in the care of the patient has suspicions as to the cause of the patient’s lung disease, it is valuable for the radiologist to be made aware of them prior to the MDD. For example, laboratory tests may reveal the presence of autoantibodies suggestive of an autoimmune disease (e.g., antinuclear antibodies, anti-cyclic citrullinated peptide, rheumatoid factor) [1, 11], while serologic testing for IgG antibodies against potential antigens can provide supportive evidence for hypersensitivity pneumonitis [9, 27]. It is important that clinicians perform a thorough patient interview to ascertain exposures to potential inducers of hypersensitivity pneumonitis such as avian antigens or microbial agents, as chronic hypersensitivity pneumonitis may have a similar clinical and radiological presentation to IPF [9, 27, 28]. Environmental exposures that increase the risk of ILD include asbestos, metal or wood dust, and farming [1]. A clinical judgment may need to be made as to whether exposure to such factors is the cause of the ILD, or simply a background exposure in a patient who has developed an idiopathic interstitial pneumonia. Information on any prescribed medications should also be provided to the radiologist. Therapies that have been associated with a UIP-like pattern of pulmonary toxicity include chemotherapeutic agents, antiarrhythmic drugs, and immunosuppressive agents [29].

**Table 1:** Factors to be considered in making a differential diagnosis in patients with suspected ILD

Age
Gender
Signs and symptoms
Inspiratory “Velcro” crackles or “squeaks” on chest auscultation
Involvement of other organs that may indicate autoimmune disease
Pulmonary function tests (PFTs)
FVC, DLco, FEV_1_
Laboratory tests that may indicate autoimmune disease or hypersensitivity pneumonitis
Occupational/environmental exposures
Smoking
Potential inducers of hypersensitivity pneumonitis e.g., birds
Exposures to compounds known to cause ILD e.g., asbestos, metal dust
Response/non-response to therapies used to treat lung disease
Use of medications known to cause ILD
Family history
Features on HRCT
Features on surgical lung biopsy, if available

The typical presentation of IPF is a male ex-smoker over the age of 50 years who presents with chronic exertional dyspnea and cough and has “Velcro”-like bibasilar inspiratory crackles on auscultation of the chest [1, 30, 31]. In patients with IPF, pulmonary function tests (PFTs) typically demonstrate a restrictive pattern (i.e., reduced total lung capacity, FVC, and diffusing capacity for carbon monoxide [DL_CO_] with a normal ratio of forced expiratory volume in one second [FEV_1_] to FVC) [32, 33]. However, it should be noted that some patients with IPF have an almost normal FVC% predicted early in the course of their disease [34] and that patients who began with an FVC of over 100% predicted may have lost a substantial amount of lung function and still have an FVC% predicted that appears normal. Further, it should be remembered that concomitant emphysema, which is present in about a third of patients with IPF, increases FVC [35].

A family history of pulmonary fibrosis should prompt consideration of familial interstitial pneumonia, although this is very rare [1]. Several mutations have been associated with an increased risk of IPF including those in genes for surfactant proteins (SFTPA2, SFTPC), telomerase reverse transcriptase (TERT), the RNA component of telomerase (TERC), and TOLLIP and MUC5B, which play important roles in lung host defense [36, 37]. Interestingly, there is emerging evidence that different polymorphisms may be associated with different patterns of fibrosis on HRCT [38].

## Patient Case Studies

### Patient Case 1

**Fig. 1:**
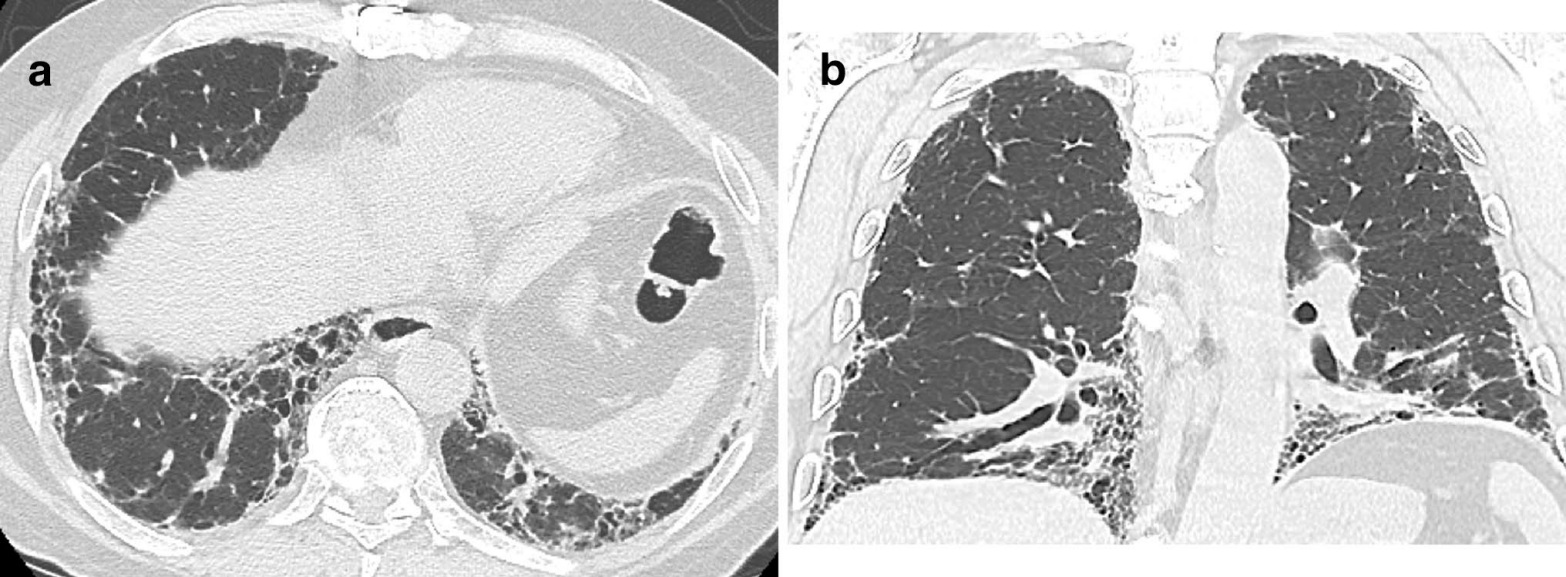
Axial (**a**) and coronal (**b**) HRCT images show peripheral and basilar predominant pulmonary fibrosis characterized by reticulation, traction bronchiectasis, traction bronchiolectasis and subpleural honeycombing consistent with UIP

A 72-year-old man presented with chronic cough, dyspnea on exertion and basilar crackles on chest auscultation. Comorbidities included coronary artery disease, hypertension, and type 2 diabetes. He was a current smoker. PFTs showed restrictive physiology (DL_CO_ of 50% predicted and FVC of 65% predicted). An HRCT scan showed definite features of UIP (Fig. 1). A consensus diagnosis of IPF was made based on the characteristic presentation in combination with definite features of UIP on HRCT and exclusion of other known causes of UIP. A lung biopsy was regarded as unnecessary and was not performed.

### Patient Case 2

**Fig. 2:**
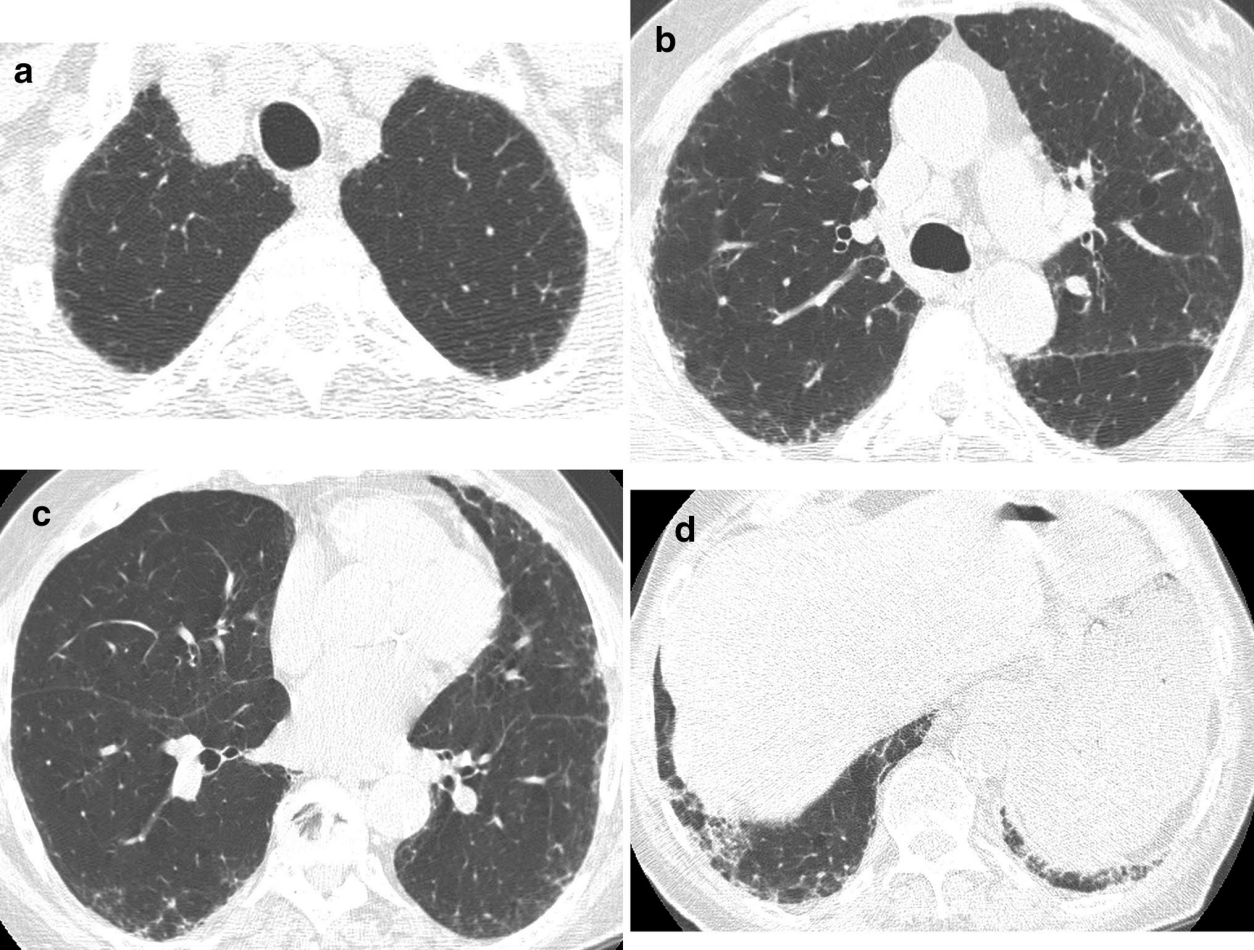
Axial (**a**–**d**) HRCT images show peripheral and basilar predominant pulmonary fibrosis characterized by reticulation, traction bronchiectasis, and traction bronchiolectasis but no honeycombing, consistent with a possible UIP pattern

A 48-year-old woman presented with chronic dyspnea, mild cough, and mild basilar crackles and “squeaks” on chest auscultation. She was a former smoker (10 pack-year history) and had a significant exposure history for parakeets, dogs, and guinea pigs. She also had a history of asthma and arthritis. PFTs showed a DL_CO_ of 65% predicted and FVC of 62% predicted. An HRCT scan showed possible UIP (Fig. 2) but the patient’s age (< 50 years) and clinical history suggested an alternative diagnosis than IPF. A consensus diagnosis of hypersensitivity pneumonitis was made given the patient’s exposures and the fact that hypersensitivity pneumonitis can present with imaging features similar to UIP.

### Patient Case 3

**Fig. 3:**
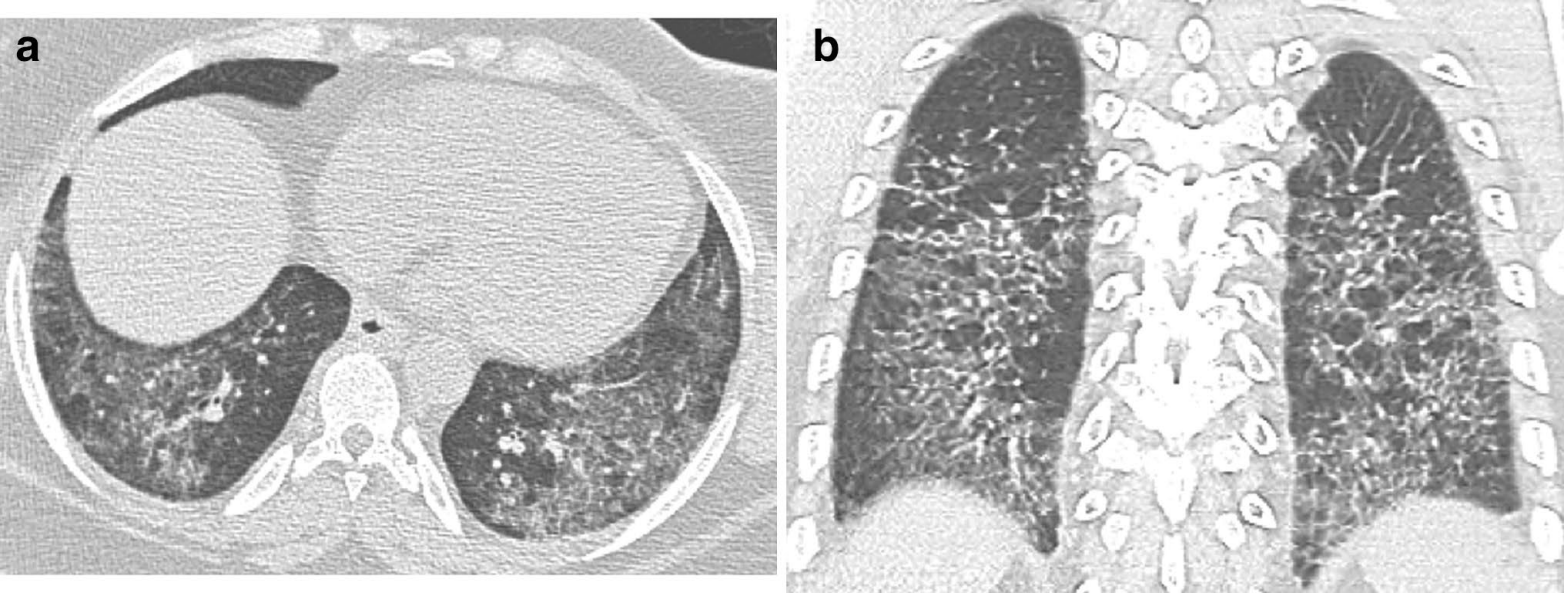
Axial (**a**) and coronal (**b**) HRCT images show basilar predominant ground-glass opacity, mild reticulation, mild traction bronchiectasis, and areas of relative subpleural sparing in the lower lobes. These findings are highly suggestive of non-specific interstitial pneumonia (NSIP)

A 67-year-old woman presented with chronic cough, fatigue, and mild basilar crackles on chest auscultation. She was a current smoker with a 20 pack-year history. Exposure history was significant for cats. She had a history of gastroesophageal reflux disease, hypertension, and migraines and comorbidities including back pain, and intermittent constipation and diarrhea. PFTs revealed a DL_CO_ of 60% predicted and FVC of 70% predicted. Laboratory findings were mildly positive for antinuclear antibody and C-reactive protein. An HRCT scan was inconsistent with UIP and most suggestive of a non-specific interstitial pneumonia pattern (Fig. 3). However, a working diagnosis of IPF was made given that the patient’s clinical history and physical examination were suggestive of IPF and no alternative cause for the patient’s interstitial pneumonia was identified. After 2 years of follow-up, the patient had joint pain and was positive for anti-cyclic citrullinated peptide and rheumatoid factor, and was diagnosed with rheumatoid arthritis.

## Conclusions

Central to the diagnosis of ILD is an HRCT scan performed and interpreted by an expert radiologist. However, the likelihood that specific radiologic features reflect IPF is dependent on the clinical context. Although both radiologists and pulmonologists can identify findings that suggest an ILD, neither can make a differential diagnosis without input from the other. In cases where the diagnosis is not clear based on the patient’s radiological and clinical features, MDD is required to agree on the most appropriate diagnosis. For some patients, a working diagnosis is appropriate, but this should be reviewed at regular intervals as new information pertinent to the patient’s diagnosis may become available over time. Diagnostic guidelines for IPF will be revised in future to reflect our growing knowledge of the clinical course of patients with specific clinical and radiological features, as well as new information on molecular signatures that predict UIP.

## References

[1] Raghu G, Collard HR, Egan JJ, Martinez FJ, Behr J, Brown KK (2011). An official ATS/ERS/JRS/ALAT statement: idiopathic pulmonary fibrosis: evidence-based guidelines for diagnosis and management. Am J Respir Crit Care Med.

[2] Raghu G, Chen SY, Hou Q, Yeh WS, Collard HR (2016). Incidence and prevalence of idiopathic pulmonary fibrosis in US adults 18–64 years old. Eur Respir J.

[3] Richeldi L, du Bois RM, Raghu G, Azuma A, Brown KK, Costabel U (2014). Efficacy and safety of nintedanib in idiopathic pulmonary fibrosis. N Engl J Med.

[4] Raghu G, Rochwerg B, Zhang Y, Garcia CA, Azuma A, Behr J (2015). An official ATS/ERS/JRS/ALAT clinical practice guideline: treatment of idiopathic pulmonary fibrosis. An update of the 2011 clinical practice guideline. Am J Respir Crit Care Med.

[5] King TE, Bradford WZ, Castro-Bernardini S, Fagan EA, Glaspole I, Glassberg MK (2014). A phase 3 trial of pirfenidone in patients with idiopathic pulmonary fibrosis. N Engl J Med.

[6] Fisher M, Nathan SD, Hill C, Marshall J, Dejonckheere F, Thuresson PO (2017). Predicting life expectancy for pirfenidone in idiopathic pulmonary fibrosis. J Manag Care Spec Pharm.

[7] Copley SJ, Wells AU, Sivakumaran P, Rubens MB, Lee YC, Desai SR (2003). Asbestosis and idiopathic pulmonary fibrosis: comparison of thin-section CT features. Radiology.

[8] American Thoracic Society; European Respiratory Society (2002). American Thoracic Society/European Respiratory Society International Multidisciplinary Consensus Classification of the Idiopathic Interstitial Pneumonias. This joint statement of the American Thoracic Society (ATS), and the European Respiratory Society (ERS) was adopted by the ATS board of directors, June 2001 and by the ERS Executive Committee, June 2001. Am J Respir Crit Care Med.

[9] Salisbury ML, Myers JL, Belloli EA, Kazerooni EA, Martinez FJ, Flaherty KR (2017). Diagnosis and treatment of fibrotic hypersensitivity pneumonia. Where we stand and where we need to go. Am J Respir Crit Care Med.

[10] Travis WD, Costabel U, Hansell DM, King TE, Lynch DA, Nicholson AG (2013). An official American Thoracic Society/European Respiratory Society statement: update of the international multidisciplinary classification of the idiopathic interstitial pneumonias. Am J Respir Crit Care Med.

[11] Doyle TJ, Dellaripa PF (2017). Lung manifestations in the rheumatic diseases. Chest.

[12] Lynch DA, Sverzellati N, Travis WD, Brown KK, Colby TV, Galvin JR (2017). Diagnostic criteria for idiopathic pulmonary fibrosis: a Fleischner Society White Paper. Lancet Respir Med.

[13] Chung JH, Lynch DA (2016). The value of a multidisciplinary approach to the diagnosis of usual interstitial pneumonitis and idiopathic pulmonary fibrosis: radiology, pathology, and clinical correlation. AJR Am J Roentgenol.

[14] Fell CD, Martinez FJ, Liu LX, Murray S, Han MK, Kazerooni EA (2010). Clinical predictors of a diagnosis of idiopathic pulmonary fibrosis. Am J Respir Crit Care Med.

[15] Hunninghake GW, Lynch DA, Galvin JR, Gross BH, Müller N, Schwartz DA (2003). Radiologic findings are strongly associated with a pathologic diagnosis of usual interstitial pneumonia. Chest.

[16] Raghu G, Mageto YN, Lockhart D, Schmidt RA, Wood DE, Godwin JD (1999). The accuracy of the clinical diagnosis of new-onset idiopathic pulmonary fibrosis and other interstitial lung disease: a prospective study. Chest.

[17] Johkoh T, Müller NL, Cartier Y, Kavanagh PV, Hartman TE, Akira M (1999). Idiopathic interstitial pneumonias: diagnostic accuracy of thin-section CT in 129 patients. Radiology.

[18] Johkoh T, Müller NL, Colby TV, Ichikado K, Taniguchi H, Kondoh Y (2002). Nonspecific interstitial pneumonia: correlation between thin-section CT findings and pathologic subgroups in 55 patients. Radiology.

[19] Lynch DA, Godwin JD, Safrin S, Starko KM, Hormel P, Brown KK (2005). High-resolution computed tomography in idiopathic pulmonary fibrosis: diagnosis and prognosis. Am J Respir Crit Care Med.

[20] Silva CI, Müller NL, Lynch DA, Curran-Everett D, Brown KK, Lee KS (2008). Chronic hypersensitivity pneumonitis: differentiation from idiopathic pulmonary fibrosis and nonspecific interstitial pneumonia by using thin-section CT. Radiology.

[21] Sumikawa H, Johkoh T, Fujimoto K, Ichikado K, Colby TV, Fukuoka J (2012). Usual interstitial pneumonia and nonspecific interstitial pneumonia: correlation between CT findings at the site of biopsy with pathological diagnoses. Eur J Radiol.

[22] Tsubamoto M, Müller NL, Johkoh T, Ichikado K, Taniguchi H, Kondoh Y (2005). Pathologic subgroups of nonspecific interstitial pneumonia: differential diagnosis from other idiopathic interstitial pneumonias on high-resolution computed tomography. J Comput Assist Tomogr.

[23] Flaherty KR, King TE, Raghu G, Lynch JP, Colby TV, Travis WD (2004). Idiopathic interstitial pneumonia: what is the effect of a multidisciplinary approach to diagnosis?. Am J Respir Crit Care Med.

[24] Walsh SL, Wells AU, Desai SR, Poletti V, Piciucchi S, Dubini A (2016). Multicentre evaluation of multidisciplinary team meeting agreement on diagnosis in diffuse parenchymal lung disease: a case-cohort study. Lancet Respir Med.

[25] Hansell DM, Bankier AA, MacMahon H, McLoud TC, Müller NL, Remy J (2008). Fleischner society: glossary of terms for thoracic imaging. Radiology.

[26] Wuyts WA, Cavazza A, Rossi G, Bonella F, Sverzellati N, Spagnolo P (2014). Differential diagnosis of usual interstitial pneumonia: when is it truly idiopathic?. Eur Respir Rev.

[27] Vasakova M, Morell F, Walsh S, Leslie K, Raghu G (2017). Hypersensitivity pneumonitis: perspectives in diagnosis and management. Am J Respir Crit Care Med.

[28] Morell F, Villar A, Montero M, Muñoz X, Colby TV, Pipvath S (2013). Chronic hypersensitivity pneumonitis in patients diagnosed with idiopathic pulmonary fibrosis: a prospective case-cohort study. Lancet Respir Med.

[29] Schwaiblmair M, Behr W, Haeckel T, Märkl B, Foerg W, Berghaus T (2012). Drug induced interstitial lung disease. Open Respir Med J.

[30] Behr J, Kreuter M, Hoeper MM, Wirtz H, Klotsche J, Koschel D (2015). Management of patients with idiopathic pulmonary fibrosis in clinical practice: the INSIGHTS-IPF registry. Eur Respir J.

[31] Cottin V, Cordier JF (2012). Velcro crackles: the key for early diagnosis of idiopathic pulmonary fibrosis?. Eur Respir J.

[32] Alhamad EH, Lynch JP, Martinez FJ (2001). Pulmonary function tests in interstitial lung disease: what role do they have?. Clin Chest Med.

[33] Egan JJ, Martinez FJ, Wells AU, Williams T (2005). Lung function estimates in idiopathic pulmonary fibrosis: the potential for a simple classification. Thorax.

[34] Kolb M, Richeldi L, Behr J, Maher TM, Tang W, Stowasser S (2017). Nintedanib in patients with idiopathic pulmonary fibrosis and preserved lung volume. Thorax.

[35] Cottin V (2013). The impact of emphysema in pulmonary fibrosis. Eur Respir Rev.

[36] Kropski JA, Lawson WE, Young LR, Blackwell TS (2013). Genetic studies provide clues on the pathogenesis of idiopathic pulmonary fibrosis. Dis Model Mech.

[37] Noth I, Zhang Y, Ma SF, Flores C, Barber M, Huang Y (2013). Genetic variants associated with idiopathic pulmonary fibrosis susceptibility and mortality: a genome-wide association study. Lancet Respir Med.

[38] Chung JH, Peljito AL, Chawla A, Talbert JL, McKean DF, Rho BH (2016). CT imaging phenotypes of pulmonary fibrosis in the MUC5B promoter site polymorphism. Chest.

